# When Intracoronary Anatomy is Superior to Physiology

**DOI:** 10.31083/j.rcm2307251

**Published:** 2022-07-14

**Authors:** Ned Premyodhin, Morton J. Kern, Arnold H. Seto

**Affiliations:** ^1^Department of Medicine, Veterans Administration Long Beach Health Care System, Long Beach, CA 90822 USA; ^2^Department of Medicine, University of California, Irvine, Orange, CA 92868, USA

**Keywords:** coronary hemodynamics, FFR, CABG, CAD

## Abstract

Physiologic assessment has become an essential tool to guide revascularization decisions due to the multiple limitations of angiographic and anatomic measures of physiologic significance. However, in certain cases the apparent physiologic measurement may not accurately reflect the severity of coronary disease compared with anatomical measurements. This article will review how anatomy trumps physiology in cases of acute coronary syndromes, left main disease, saphenous vein graft lesions, and myocardial bridging, and how to overcome the limitations of physiologic measurement in these clinical situations.

## 1. Introduction

Angiographic anatomy has been the standard for the assessment of coronary artery disease since its inception in 1964 by Mason Sones of the Cleveland Clinic. However, in the quantitation of specific ischemia-producing lesions, angiography fails. It falls short in attempting to translate the three-dimensional artery stenosis morphology from two-dimensional “lumenograms” into meaningful physiology. Even precise quantification of stenosis severity by computer assisted quantitative coronary angiography (QCA), a technique more accurate than two- or three-dimensional resolution of coronary luminograms, cannot produce a clinically useful prediction of coronary physiology associated with ischemia [1]. Improvements in coronary computer tomography as well as in-lab three-dimensional angiographic vessel reconstruction can generate fractional flow reserve (FFR) maps of the vessels. However, the in-lab angiographically derived FFR is undergoing trials and has not yet become incorporated into the daily cardiac catheterization (cath) lab practice [2].

Direct guidewire-based measurements of intracoronary blood flow and pressure provide unique information that complements the angiographic (i.e., anatomic) evaluation and facilitates better decision-making regarding the ischemic risk to guide therapy [3]. The application of this technology with improved sensor angioplasty guidewires has expanded to numerous clinical scenarios beyond the simple functional assessment of intermediate lesions to more complex scenarios. Despite this diffusion of in-lab ischemia tests with physiology, there remain a few important anatomic and clinical scenarios where physiologic testing is questioned, and where anatomic considerations trump physiology in patient management decisions. These issues will be reviewed in this chapter.

## 2. Considerations for Anatomy Over Physiology

In this chapter, anatomy will refer to any modality which can display the coronary artery or coronary stenosis using either angiography (invasive or non-invasive) or intravascular imaging with ultrasound (IVUS) or optical coherence tomography (OCT). When referring to physiology in general, it implies the use of either hyperemic translesional pressure measures (FFR) or non-hyperemic pressure ratios (NHPR, such as iFR, Pd/Pa, dPR, DPR, RFR, etc.) or any measure of coronary blood flow or resistance. Specific applications for one method over another will be addressed in the appropriate context.

Anatomy may trump physiology when (1) the physiologic measurement accuracy is questioned, (2) the clinical presentation is associated with dynamically changing coronary blood flow (e.g., during ST-segment elevation myocardial infarction [STEMI]), or (3) the complexity of anatomy makes it impossible to assess the physiology of individual lesions such as may occur in multiple lesions in series or diffuse disease. Table 1 lists considerations for use of intravascular imaging over invasive physiologic assessment indices.

**Table 1.** **Common cases where physiologic assessment is limited. Listing of conditions where the use of either anatomic or physiologic assessment may be appropriate**.

	Favors anatomic assessment	Favors physiologic assessment	Comments
Acute coronary syndrome	Microvascular bed subtended by non-IRA is in close proximity to IRA	Non-IRA in distant microvascular bed	Positive/abnormal FFR in non-IRA stenosis is reliable
	<24–48 hours of ACS in IRA	>4–6 days after ACS in IRA	Negative/abnormal FFR in non-IRA may be falsely negative
	Negative FFR in non-IRA may be falsely negative	abnormal FFR in non-IRA stenosis is reliable	Non-culprit TCFA with high atherosclerotic plaque burden ≥70% or MLA of ≤4 mm are higher risk for recurrent events
Left main disease	LM disease with downstream LCX and LAD disease	Isolated LM disease	If the FFR epicardial (LM+LAD) is <0.60, the apparent LM FFR in the CFX will potentially be falsely negative
	LM disease with severe downstream disease with combined FFR ≤0.60	LM disease with significant disease in LCX or LAD when combined FFR ≥0.60	In these situations, an intravascular ultrasound assessment of the LM with a threshold minimal luminal area of <6.0 ${\mathrm{mm}}^{2}$ is recommended
Saphenous vein grafts	Presence of distal collaterals with variable native vessel obstruction	Absence of distal collaterals and FFR of native vessel + SVG <0.80 suggestive of potential ischemia	OCT of culprit lesions in old SVGs shows thin fibrous cap, plaque rupture and thrombus with increasing evidence of thrombus in myocardial infarction than unstable angina
			Limited anatomic parameters of SVG lesions available to guide intervention
Myocardial bridging	Negative FFR or iFR with clinical presentation or angiography concerning for ischemia in myocardial bridging	Significant positive FFR or iFR findings	IVUS characteristic findings include an echolucent band partially or completely encircling target artery
		Significant plaque burden within or immediately proximal to the myocardial bridge	Cross-sectional area, external elastic membrane and plaque burden within and proximal to bridge correlate with degree of arterial compression
Cardiac allograft vasculopathy	Conventional angiography is the norm for surveillance of CAV although is limited in detection of early intimal disease	Significant donor transmitted atherosclerosis	FFR <0.90 and IMR >20 measured in the proximal LAD 1 year after transplant correlate with worse outcomes
	IVUS can detect early changes by quantitating intimal medial changes	FFR and indexes of microcirculatory resistance characterize vasomotor dysfunction in CAV	Volumetric IVUS demonstrating early changes in intimal medial volume in the proximal LAD associated with worse outcomes
Optimizing post-PCI FFR	Post-PCI FFR <0.90; iFR <0.95	Post-PCI FFR of >0.90; iFR >0.95 associated with optimal stent expansion and improved outcomes	Minimal stent area can be measured by anatomic imaging while guiding optimization of stent deployment
	Concern for stent under expansion, malapposition, dissection or plaque protrusion	Downstream lesion of questionable significance	Degree of stent expansion not significantly different by IVUS-guided or OCT-guided PCI
	Anatomically challenging lesions		

Abbreviations: ACS, acute coronary syndrome; CAV, coronary allograft vasculopathy; FFR, fractional flow reserve; iFR, instantaneous wave-free ratio; IMR, index of microcirculatory resistance; IRA, infarct related artery; non-IRA, non-infarct related artery; IVUS, intravascular ultrasound; LAD, left anterior descending coronary artery; LCX, left circumflex coronary artery; LM, left main coronary artery; MLA, minimum luminal area; OCT, optical coherence tomography; SVG, saphenous vein graft; TCFA, thin-cap fibroatheromas.

The techniques to obtain best accuracy of physiologic measurements in the cath lab have been addressed in detail elsewhere [4, 5]. Accurate measurements require attention to tubing and electrical connections, bubble/blood free lines, correct zeroing/calibrations, and standard dosing and administration of adenosine or other hyperemic agents.

## 3. Common Clinical Scenarios 

The most common clinical scenarios when anatomy trumps physiology include the acute coronary syndromes, left main stenosis with downstream disease, saphenous vein bypass lesions, myocardial bridging and allograft vasculopathy. The best results following stent implantation requires visualization of full strut apposition and expansion and requires intracoronary imaging which physiology cannot provide. Nonetheless, post-percutaneous coronary intervention (PCI) physiology will be informative about residual, hidden, or diffuse disease detection. In some cases, such as lesion assessment prior to coronary artery bypass grafting, the superiority of physiologic testing has not been clearly established or accepted and anatomy is still commonly used to guide treatment.

## 4. Acute Coronary Syndromes

In acute coronary syndromes (ACS), especially in acute STEMI, the pathophysiology of the infarcted artery and its subtended and infarcted or damaged microvascular bed is both dynamic and complex. The ability of FFR to detect ischemia for either the culprit or non-culprit (i.e., the non-infarct related artery, non-IRA or NIRA) in ACS has several limitations: (1) the microvascular bed in the infarct zone may not have uniform, constant, or minimal resistance; (2) the hemodynamic severity of stenosis of the infarct related artery, IRA, may evolve during the recuperative phase as occlusive thrombus and vasoconstriction abate; and (3) in ACS, FFR measurements are not meaningful when normal perfusion has not been achieved. Thus, FFR has limited utility in the IRA during the first 24–48 hours after a STEMI or non-ST segment elevation myocardial infarction (NSTEMI). In contrast, FFR has demonstrated value in the non- IRAs [6] with increasing confidence as the distance between the culprit territory and the non-IRA territory becomes greater. Territories remote from the injury area have more stable myocardial flow and hence more reliable translesional physiology. Physiology of a presumed culprit lesion becomes reliable >2–4 days during the recovery phase of acute MI.

**Fig. 1.**
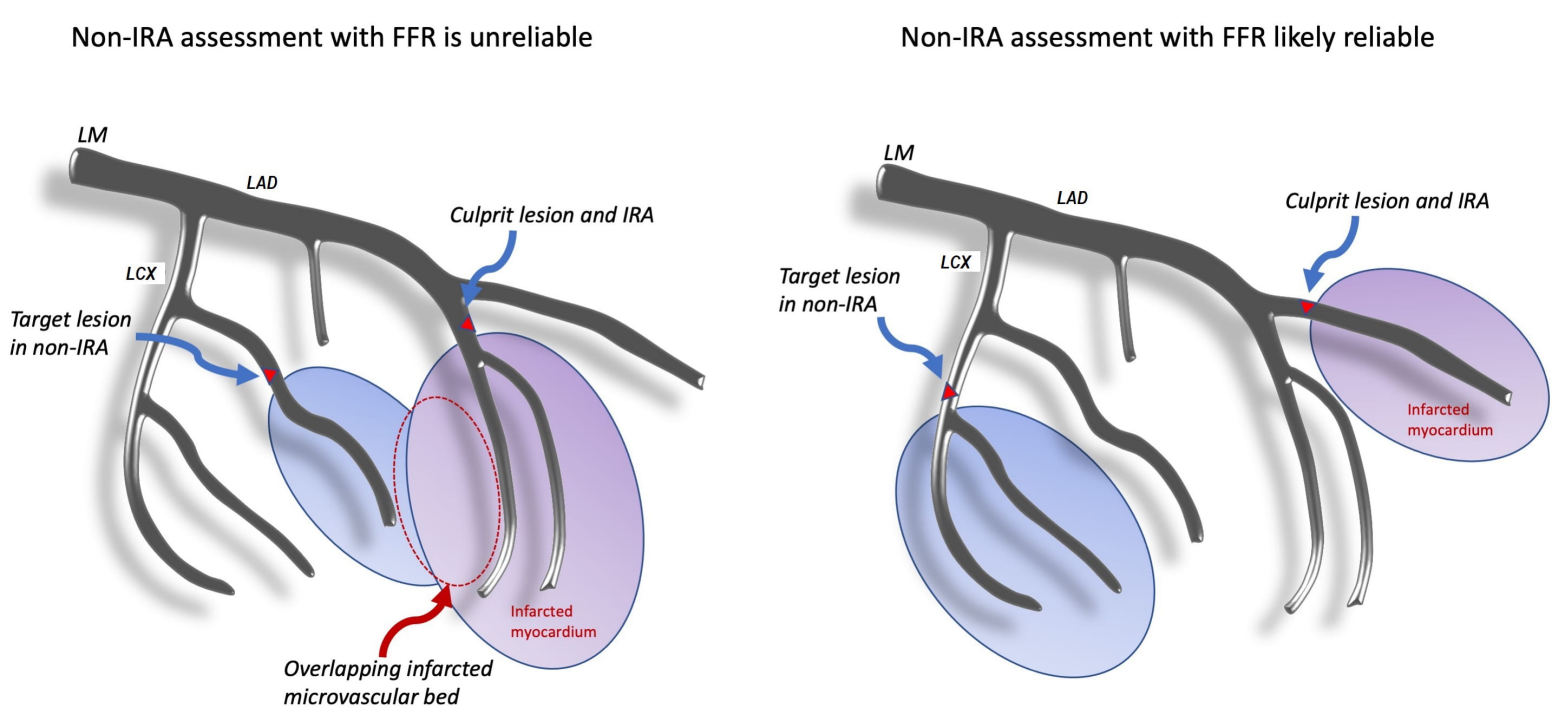
**Anatomic dependence of non-IRA assessment in acute coronary syndromes with zones of perfusion and infarcted myocardium**. Overlapping infarcted microvascular bed represents a source of error in non-IRA FFR. Abbreviations: FFR, fractional flow reserve; LAD, left anterior descending coronary artery; LCx, left circumflex coronary artery; LM, left main coronary artery; IRA, infarct related artery; non-IRA, non-infarct related artery.

In the infarct zone during the acute phase, myocardial blood flow is reduced and FFR may be falsely elevated due to the lower total flow (Fig. 1). For this reason, physiology is not reliable in the STEMI culprit artery until 4–6 days after the event, when myocardial function is believed to stabilize and achieve its normal maximal flow capabilities.

For the non-IRA, in STEMI/NSTEMI patients, the exact borders of the zone of myocardial injury from the culprit vessel is unknown but may extend close to the region supplied by the non-IRA. As a result, a normal non-IRA FFR at the time of STEMI might be lower several days later as the coronary flow improves to the remote non-IRA zone, thus potentially changing the initial treatment decision based on a high FFR. Fortunately, however, for whatever level of flow is generated across the non-IRA stenosis, a positive abnormal result remains reliable. It is nearly impossible to have a false positive FFR barring any technical problem. Subsequently, a low FFR indicates a significant flow limiting lesion, while a high FFR may be misleadingly negative.

Complete revascularization in the STEMI/NSTEMI patient is associated with better outcomes. Failure to address the non-culprit vessels whether at the same setting or staged, results in higher rates of heart failure, recurrent ACS and the need for further revascularization with lower survival. The PRIMULTI study demonstrated that at 2 years, major adverse cardiac events occurred in 22% of patients who received culprit-only PCI of the STEMI vessel but only in 13% of participants in the FFR-guided revascularization of all significant, non-infarct related arteries (Hazard Ratio (HR) 0.56, *p* = 0.004) [7]. Therefore, reliable assessment of non-culprit lesions within the acutely infarcted microvascular bed would influence decisions to treat at the time of primary PCI and improve outcomes. Ntalianis *et al*. [6] demonstrated that FFR of non-culprit lesions is reliable and accurate when comparing values at the index procedure to those at 3 month follow up. Nonetheless, variations in individual anatomy and proximity of non-culprit lesions to the infarcted microvascular bed will play a role in the clinical usefulness of pressure measurements.

In ACS patients, anatomic assessment with intravascular imaging may be considered to improve prognosis. In the PROSPECT study, 697 patients with ACS underwent IVUS of culprit and non-culprit vessels after primary PCI and the cumulative rate of major adverse cardiovascular events after 3 years was monitored. Non-culprit lesions that were classified as thin-cap fibroatheromas (HR 3.35, *p *< 0.001), had high atherosclerotic plaque burden ≥70% (HR 5.03, *p *< 0.001), or minimal luminal area of ≤4 ${\mathrm{mm}}^{2}$ (HR 3.2, *p *< 0.001) were more likely to be associated with recurrent events than non-culprit lesions that did not exhibit these properties [8]. While the IVUS findings were not used to guide therapy, this study highlights morphologic findings that may have clinical utility in identifying high risk non-culprit lesions that should be treated. In a retrospective cohort study, IVUS-guided PCI during acute myocardial infarction (AMI) was also associated with a lower rate of major adverse cardiac event (MACE) at 1 year and beyond compared to angiography-guided PCI only (HR 0.766, 95% CI: 0.650–0.903, *p* = 0.002) in propensity matched analysis [9]. This benefit is largely attributed to stent selection and optimization, with subgroup analyses pointing towards a greater benefit in patients with CKD. Although further investigation is needed before IVUS can be used to guide preventive stenting, anatomic assessment can provide valuable information when the accuracy of FFR is in question.

## 5. Left Main Stenosis

Accurate assessment of the hemodynamic significance of left main coronary lesions (LM) is critical for patient decision making for medical therapy, PCI or coronary artery bypass grafting (CABG) surgery. FFR has been used to assess intermediate LM lesions, particularly in cases of isolated LM lesions or LM lesions with significant disease in either the left circumflex coronary artery (LCX) or left anterior descending coronary artery (LAD). Fearon *et al*. [10] demonstrated that although the apparent FFR of intermediate LM lesions measured in a non-diseased LCX or LAD is elevated when there is downstream disease in the other, the magnitude of this effect is rarely clinically significant unless the combined FFR of the LM and LAD is ≤0.60. However, in the complex case where both the LAD and LCX have significant downstream disease, FFR for the LM was not deemed accurate and anatomic imaging became the tool of choice. Cases of left main disease with significant LCX and LAD disease present a challenge to physiologic assessment wherein anatomy with intravascular imaging modalities may provide more reliable information than FFR [11]. Furthermore, aorto-ostial lesions create unique challenges for the use of pressure-derived FFR due to the requirement to disengage the guide catheter and inability to administer intracoronary adenosine.

Physiology is often favored for assessment of simple, isolated LM stenosis or distal LM bifurcation stenosis which can be easily assessed with two FFR/NHPR measurements, one in the LAD and another with the pressure wire in the CFX. However, interpreting the LM FFR in the presence of significant downstream branch lesions is more complicated because the LM and LAD/CFX lesions behave like serial lesions. The true flow across the LM is potentially reduced by a severe downstream stenosis, artifactually elevating the LM FFR when measured in the unobstructed vessel.

**Fig. 2.**
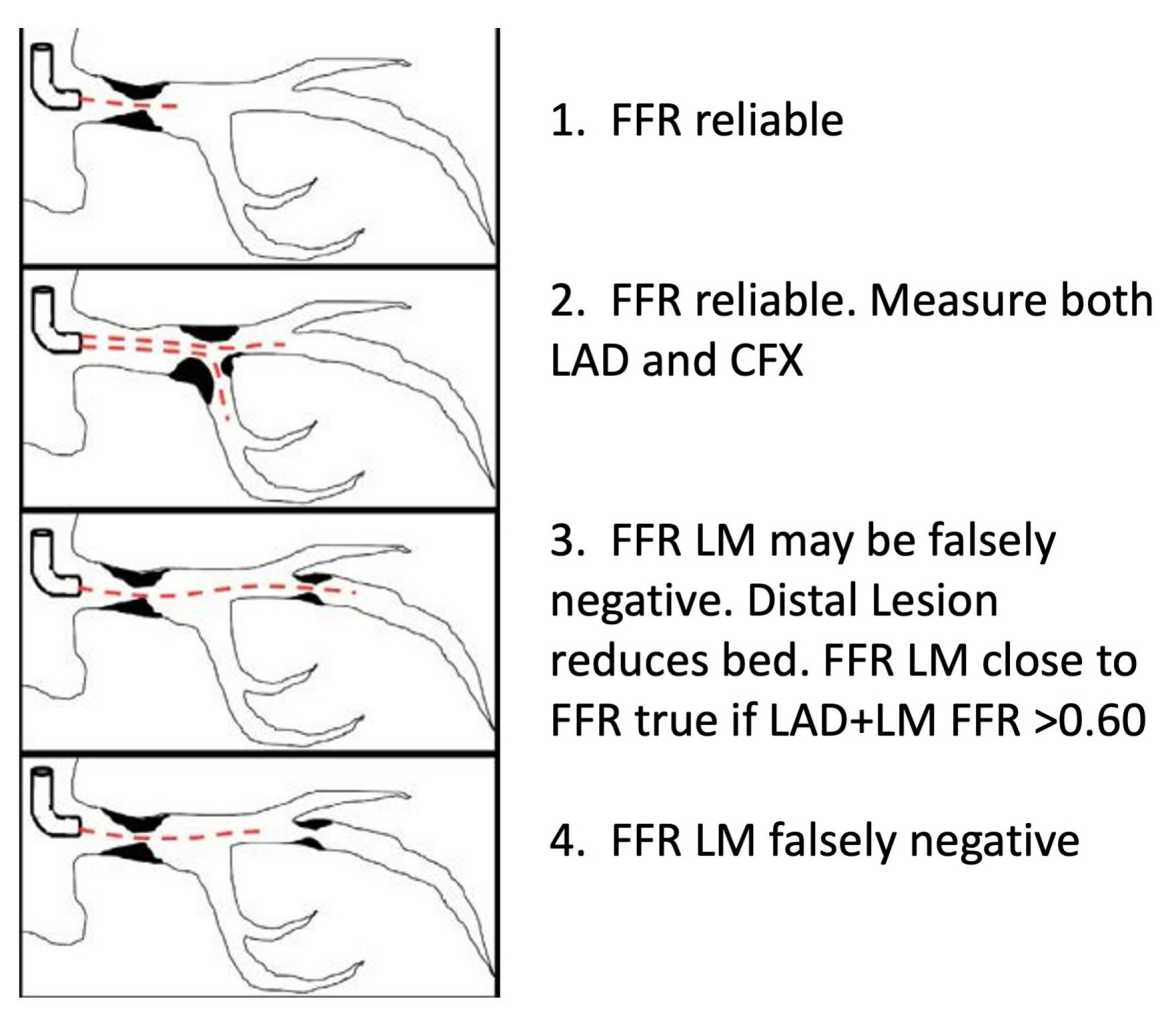
**FFR of left main stenosis with downstream disease**. FFR is reliable in isolated LM disease and distal LM disease with ostial involvement when FFR is measured across both LAD and LCx. LM FFR may be falsely negative when FFR of LAD+LM together fall below 0.60 or when significant downstream disease is present but FFR is measured across only the LM lesion. Abbreviations: FFR, fractional flow reserve; LAD, left anterior descending coronary artery; LCx, left circumflex coronary artery; LM, left main coronary artery.

In this scenario, maximal hyperemia across the LM stenosis may be attenuated due to a severe LAD lesion reducing the LAD bed size (i.e., flow). Flow through the LM artery is proportional to the size of each artery’s viable myocardial bed. When LM FFR is measured in the unobstructed CFX artery, the reliability of this measurement will depend on whether the LAD stenosis is severe enough to impair flow. The lower LM flow would produce an erroneously elevated FFR because true maximal hyperemia would not be achieved (Fig. 2).

**Fig. 3.**
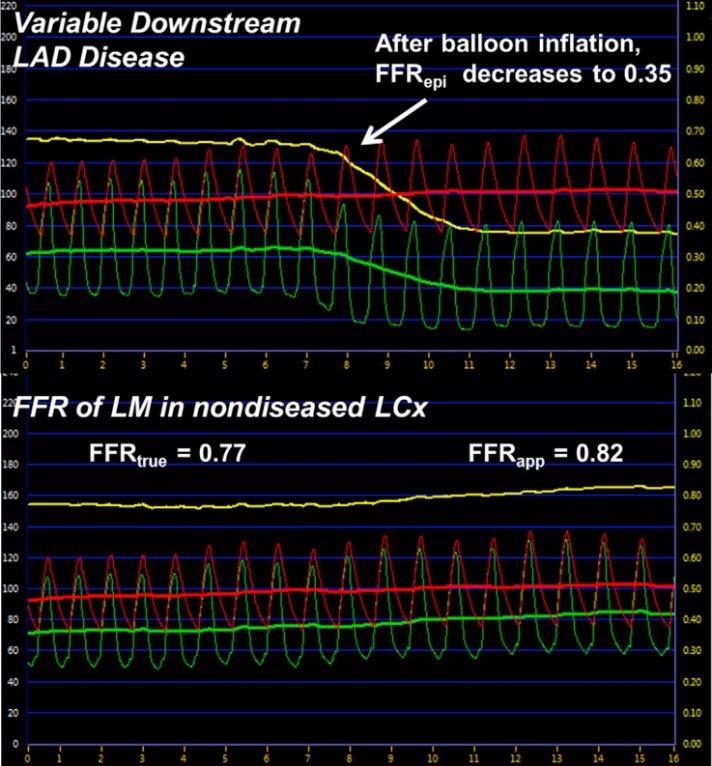
**Effects of simulated distal obstruction on LM FFR**. (Top) Coronary pressure recorded from the distal LAD as a balloon is inflated simulating variable downstream LAD disease and the effect on ${\mathrm{FFR}}_{\text{epi}}$. (Bottom) Coronary pressure recorded simultaneously as the top panel but from the LCx (representing ${\mathrm{FFR}}_{\text{true}}$ and ${\mathrm{FFR}}_{\text{app}}$) as the balloon is inflated in the LAD. Green line represents distal coronary pressure (Pd), the red line represents aortic pressure (Pa), and the yellow line is the calculated FFR value. Abbreviations: FFR, fractional flow reserve; ${\mathrm{FFR}}_{\text{app}}$, apparent FFR; ${\mathrm{FFR}}_{\text{epi}}$, epicardial FFR; ${\mathrm{FFR}}_{\text{true}}$, true FFR; LAD, left anterior descending artery; LCx, left circumflex coronary artery; LM, left main coronary artery. Taken from Fearon *et al*. JACC Cardiovasc Interv. 2015 Mar; 8(3): 398-403 available under open access.

In practice, the LM FFR in the setting of LM and LAD disease is assessed by placing the pressure wire sensor distal to the LAD lesion, administering adenosine hyperemia (either intravenously or intracoronary), and calculating the FFR across both lesions, which is called FFRepicardial. If FFRepicardial is >0.80, neither lesion is physiologically significant, and no further intervention is needed. However, if the FFRepicardial is ≤0.80, the operator can measure FFR in the CFX. An apparent LM FFR (${\mathrm{FFR}}_{\text{app}}$) in the CFX, of >0.80 indicates that the LAD, but not the LM, is hemodynamically significant. However, if the FFR epicardial (LM+LAD) is <0.60, the apparent LM FFR in the CFX will potentially be falsely negative. In these situations, an intravascular ultrasound assessment of the LM with a threshold minimal luminal area (MLA) of <6.0 ${\mathrm{mm}}^{2}$ is recommended. The reliability of the LM FFR depends on operator technique, accurate hemodynamic signal acquisition, and adequate maximal hyperemia (Fig. 3).

In addition to providing information on plaque and luminal characteristics, IVUS or OCT measurements of cross-sectional areas and lesion lengths will establish the significance of LM disease and guide the decision to intervene. In the multicenter, prospective LITRO study, an MLA of <6 ${\mathrm{mm}}^{2}$ has been identified as a safe threshold for intervention in intermediate LM lesions. 178 patients with an MLA of 6 ${\mathrm{mm}}^{2}$ or more had intervention deferred and exhibited a 97.7% cardiac death-free survival, and 87.3% event free survival after 2 years. 158 patients with an MLA <6 ${\mathrm{mm}}^{2}$ received intervention and had a 94.5% cardiac death-free survival and 80.6% event-free survival after 2 years (*p* = 0.5 and *p* = 0.3 respectively, compared to the intervention group) [12].

A retrospective Spanish study of pooled patient data including 505 participants who underwent IVUS guided LM PCI with drug eluting stents (DES) propensity-matched with 505 individuals who had PCI without IVUS guidance demonstrated that survival free of cardiac death, myocardial infarction, and target lesion revascularization at 3 years was 88.7% in the IVUS group and 83.6% in the no-IVUS group (*p* = 0.04) for the population with any LM intervention. The subgroup with distal LM exhibited 90% and 80.7% 3-year survival free of major adverse cardiac events in the IVUS and no-IVUS groups respectively (*p* = 0.03) [12]. IVUS may be a valuable tool in the assessment of complex LM lesions and identifying cases where intervention would be beneficial based on luminal anatomy.

## 6. Saphenous Vein Graft Lesions 

**Fig. 4.**
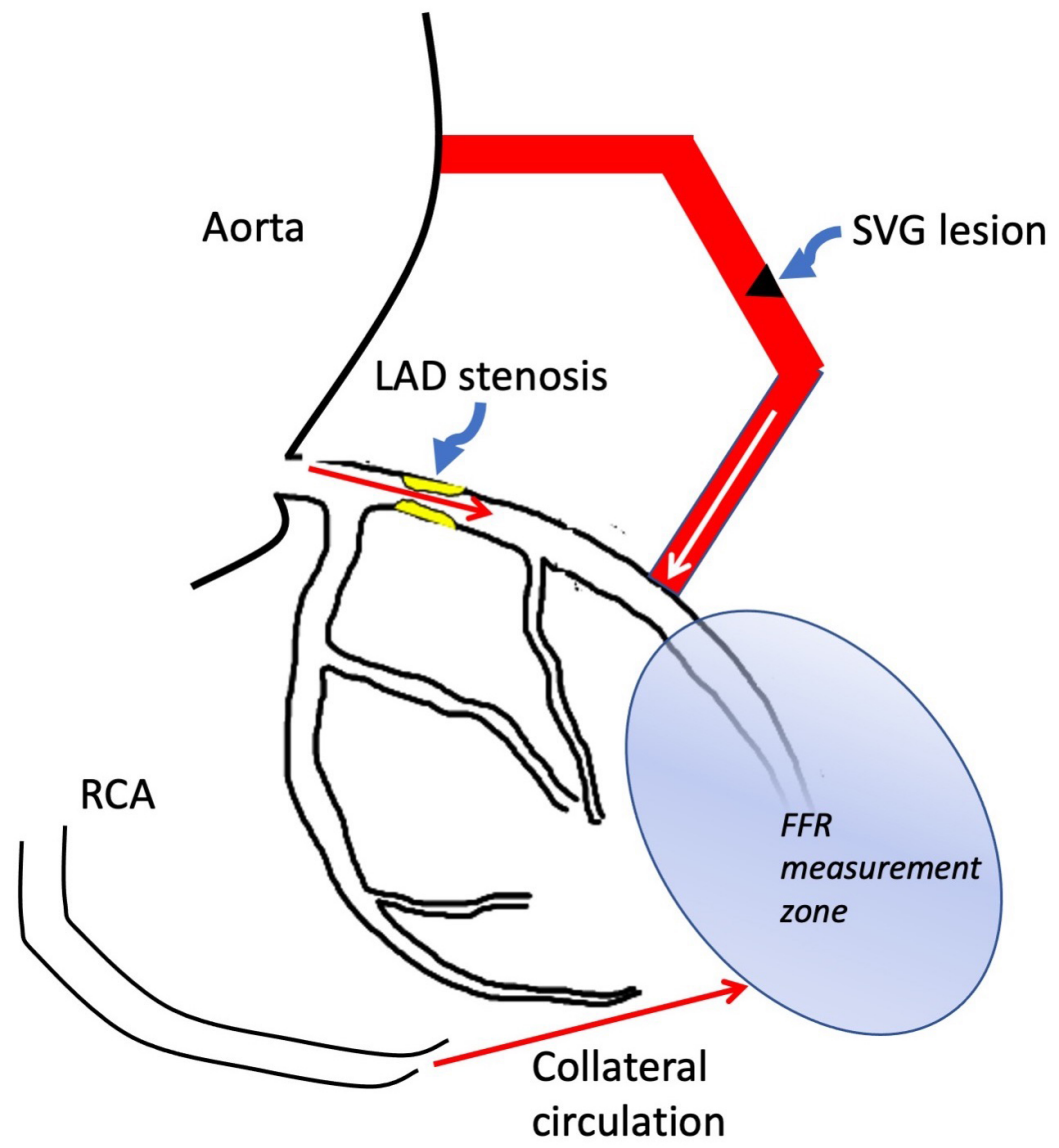
**Multiple sources of flow in the assessment of saphenous vein grafts**. FFR measured distal to the SVG attachment site reflects flow from the SVG graft, the native vessel and any collateral circulation that has formed and may be misleading in assessing the significance of SVG lesions. Abbreviations: FFR, fractional flow reserve; LAD, left anterior descending coronary artery; RCA, right coronary artery.

Saphenous vein grafts (SVGs) are susceptible to accelerated degradation compared to their arterial counterparts despite normal flow. Neointimal growth with macrophage invasion results in early atherosclerosis. Unique to SVGs is the distal myocardial bed which is perfused from 3 sources. When assessing SVG lesions, one should consider that supply to the downstream location where Pd is measured includes the native epicardial artery, the bypass conduit, and any collateral circulation that has developed. The measured FFR is thus the summed response of the of the three competing flows during maximum hyperemia. The relative contributions of each source of flow and pressure is dependent upon the extent of native vessel occlusion, the severity of stenosis within the SVG, and degree of collateralization from long-standing disease (Fig. 4). The net SVG FFR measurement indicates the potential ischemia in the region but the decision to intervene on SVG lesions must also consider the active biology of the degenerated conduit as much as the FFR before undertaking SVG PCI with the potential to accelerate graft failure.

A limited prospective study compared outcomes of deferring intervention on SVG lesions to native coronary arteries with measured FFR >0.80. 33 patients underwent FFR of SVG lesions compared to 532 who underwent native vessel FFR during the study. At a median follow-up of 3.2 years, the rate of MACE was significantly higher in the SVG group (36% versus 21%, log rank *p* = 0.01). The rate of target vessel failure was also significantly higher in the SVG group (27% versus 14%; *p* = 0.01). These findings suggest that negative FFR measurements in SVG lesions do not provide the same reassurance against MACE as for native vessel lesions [13].

Another small, underpowered study comparing FFR of SVG lesions to results of myocardial perfusion imaging in 10 patients found that the sensitivity and specificity of FFR for SVG lesions were 50% and 75%. The study also showed poor correlation between FFR and angiographic degree of stenosis for SVGs [14]. Taken together, the evidence recommends avoiding clinical decision making based on the physiologic assessment of SVG lesions and FFR has not been routinely adopted for this application.

With this background, imaging has the potential to improve decisions for intervention in SVGs. An OCT study of culprit SVG lesions in ACS found they were characterized morphologically by thin fibrous caps and fibrofatty composition. In the acute phase, thrombus was seen with increasing prevalence in NSTEMI and STEMI compared to unstable angina. 100% of culprit SVG lesions resulting in STEMI demonstrated a thin fibrous cap compared to 53.3% for NSTEMI and 20% for unstable angina (*p* = 0.03), a finding that may be useful in identifying culprit SVG lesions [15]. Anatomic intravascular imaging may play a larger or complementary role in investigating SVG lesions in the future.

## 7. Myocardial Bridging

Myocardial bridging occurs when a segment of an epicardial coronary artery traverses into the myocardium resulting in tunneling and subsequent compression by the surrounding myocardium during systole. The myocardium forms a bridge over the buried segment of the coronary artery. The extent to which symptomatic ischemia is observed depends upon the depth of the tunneled artery, the length of the tunneled segment, the number and location of affected side branches and ultimately, the degree of systolic compression. The classic finding is an angiographic systolic narrowing of the vessel (≥70% reduction in the minimal luminal diameter during systole and persistent ≥35% reduction in minimal luminal diameter during mid to late diastole), although conventional angiography has demonstrated low sensitivity in detecting myocardial bridging [16].

FFR has been used in the evaluation of myocardial bridging, however its utility in evaluating dynamic obstructions is limited. Tarantini *et al*. [17] demonstrate that following dobutamine infusion when coronary compression was maximal and patients developed ischemic changes, median FFR did not significantly change. This potentially relates to the artificial reduction in systolic pressure gradients due to “distal pressure overshooting” or the phenomenon of increased pressure measured distal to the myocardial bridge resulting in a falsely high FFR. Diastolic FFR or iFR may be more accurate in the functional assessment of a myocardial bridge but is relegated to measurements in diastole with limited assessment during systolic coronary compression.

IVUS has demonstrated the ability to measure arterial wall compression in a reproducible fashion. Characteristic findings include an echolucent band partially or completely encircling the target artery with compressive changes during systole. IVUS can also reliably measure the cross-sectional area, external elastic membrane and plaque burden within and immediately proximal to the myocardial bridge which has been shown to correlate with the degree of arterial compression [18]. In comparison, OCT can provide detailed information regarding the morphology of vulnerable plaque owing to its superior resolution, although may be limited in the detection of myocardial bridging due to limited penetration depth and rapid pullback protocol compared to IVUS [16].

## 8. Cardiac Allograft Vasculopathy (CAV)

For heart transplant vasculopathy, IVUS has been a standard by its ability to quantitate intimal medial thickening, a characteristic of early cardiac allograft vasculopathy (CAV). While conventional angiography is the norm for surveillance of CAV, it cannot detect early intimal disease or microvascular disease. It has been demonstrated that FFR of <0.90 and index of microcirculatory resistance (IMR) of ≥20 measured in the proximal left anterior descending (LAD) 1 year after transplant correlate with worse outcomes by detecting early microvascular dysfunction and severity of donor-transmitted atherosclerosis [19].

IVUS can also detect early changes in intimal medial thickness and vascular remodeling before their appearance on conventional angiography. A prospective study of 101 patients using volumetric IVUS demonstrated that paradoxical vessel remodeling characterized by intimal volume change in the proximal LAD was associated with death or need for re-transplantation [20]. Similar studies evaluating intimal medial thickness with IVUS, and OCT have proposed criteria for identifying early CAV, however standard metrics have not yet been published. Nonetheless, intravascular imaging has been an important tool for understanding the pathophysiology of and diagnosing CAV.

## 9. Anatomic Assessment in Optimizing Post-PCI FFR

Physiology plays little role in knowing the final status of a deployed stent except to test whether a post stent pressure gradient is associated with a mechanical defect (i.e., edge dissection) or whether another downstream lesion previously ignored now becomes manifest. The degree of stent expansion and apposition as defined by minimal stent area (MSA) after PCI portends the likelihood of stent thrombosis or restenosis. IVUS guidance of stent placement has been shown to be superior to angiography with reduced rates of major adverse cardiac events. Specifically, intravascular imaging can identify under expansion, malapposition, and plaque protrusion. Modern OCT and IVUS software include utilities that assist the operator in stent selection and pre-PCI planning to optimize deployment and post-PCI MSA. Since 2013, the Society of Cardiovascular Angiography and Interventions expert consensus guidelines have recommended the use of IVUS as a definitely beneficial method for determining optimal stent deployment by helping to identify complete stent expansion, apposition and edge dissection [4].

A 2016 meta-analysis of 7 trials including 3192 patients comparing outcomes of IVUS versus angiographic guidance of PCI with DES found that after 15 months, IVUS was associated with a lower risk of MACE (6.5% versus 10.3%, OR 0.60, *p *< 0.0001), cardiovascular mortality (0.5% versus 1.2%, OR 0.46 with *p* = 0.05) and stent thrombosis (0.6% versus 1.3%, OR 0.49, *p* = 0.04) [21]. Although IVUS was utilized as the initial intravascular imaging modality due to earlier introduction, OCT is gaining traction with a growing number of studies comparing their effects on outcomes.

The ILUMIEN II study compared the relative degree of stent expansion after OCT guided FFR PCI in 354 patients to the degree of stent expansion by IVUS-guided FFR PCI in 572 patients from the ADAPT-DES study using both a covariate-adjusted analysis of all participants as well as a propensity-matched pair analysis. The degree of stent expansion was not significantly different between OCT and IVUS guided FFR PCI (*p* = 0.29 in the matched-pair analysis and *p* = 0.84 in the covariate-adjusted analysis) [22]. The rapid development of new hardware and software features as well as improvements to imaging acquisition will influence operator preference and applicability of one technology over the other.

Further studies to assess whether use of IVUS guided optimization of post-PCI FFR compared to no additional intervention (standard of care) will improve MACE rates associated with post-PCI FFR of <0.90 are underway. The FFR-REACT trial is a prospective, single-center randomized controlled trial evaluating 290 patients randomized 1:1 to either IVUS or standard of care. The primary endpoint has been defined as a composite of cardiac death, target vessel re-infarction and target vessel failure requiring revascularization after 1 year. The study will also evaluate procedural success, stent thrombosis, and changes in post PCI FFR as well as physiologic and anatomic dimensions as measured by FFR and IVUS [23]. In the interim, IVUS has demonstrated benefit for optimizing stent deployment, thus should be considered when post-PCI FFR/NHPR falls below 0.90.

## 10. Anatomic versus Physiologic Guidance of Coronary Artery Bypass

Although the utility of physiologic assessment and FFR in guiding PCI has been well-established, its role in guiding lesions for bypass grafting is less clear. A number of prospective trials comparing FFR-guided to angiography guided bypass grafting have shown mixed results. The Graft Patency After FFR-Guided versus Angiography-Guided CABG (GRAFFITI) trial showed no difference in overall graft patency or MACE after 1 year [24]. However, a repeat analysis after 6 years showed a significant reduction in the rate of death or MI in the FFR-guided group (HR 0.59, 95% CI: 0.38–0.93, *p* = 0.020) [25]. Why data supporting the use of FFR-guided bypass is not as robust as compared to PCI may be due to higher complexity of lesions (including serial lesions, or diffuse epicardial disease with impaired distal microcirculation). For many surgeons, angiographic significance defined as >50% stenosis remains the threshold for bypass, and in such cases, avoidance of physiologic testing may reduce procedural time, contrast exposure and risk of native vessel injury, subsequently reducing the risk of complications during CABG. Nonetheless, the accurate assessment and identification of functionally significant lesions for bypass remains of critical importance as grafting less critical stenoses contributes to early vein or arterial graft failure from competitive flow [26]. Additional prospective studies are needed to demonstrate that functional assessment has a significant benefit in coronary bypass surgery.

## 11. Conclusions

Despite advancements in modern cardiovascular intervention, anatomic assessment will never be supplanted by physiology. Anatomy though will continue to fail in consistently demonstrating hemodynamic lesion significance. Recognizing the shortcomings of coronary pressure measurements is of particular importance when their findings influence decisions to proceed down major decision branch points of clinical management, such as the decision to refer for surgery. For the less common cases where physiology is known to fail, as highlighted above, the understanding of which imaging methods can reliably guide or optimize intervention is invaluable. Prospective comparative studies will illuminate when anatomic assessments improve outcomes as well as establish definitive parameters for use. Until then, there is a strong argument for integrating both anatomic (angiographic FFR, IVUS/OCT, and FFRCT) and physiologic assessment into standard practice.
